# Hyperpigmentation Disorders Management in India: A Delphi Based Expert Consensus on Epidemiology, Therapeutic Challenges, and the Evolving Role of Dermocosmetics

**DOI:** 10.1111/jocd.71188

**Published:** 2026-09-28

**Authors:** Mukta Sachdev, Neena Khanna, Niti Khunger, Sushil Tahiliani, Anil Ganjoo, Nina Madnani, Maya Vedamurthy, Abhishek De, Rajesh Kumar, Delphine Kerob, Davinder Prasad

**Affiliations:** ^1^ M S Clinical Research Pvt Ltd Bangalore India; ^2^ Manipal Hospital Bengaluru India; ^3^ School of Medicine Amrita Hospital Faridabad India; ^4^ VM Medical College & Safdarjang Hospital New Delhi India; ^5^ P.D. Hinduja National Hospital & MRC Mumbai India; ^6^ Private Practice—Dr Tahiliani Clinic Mumbai India; ^7^ Skinnovation Clinics New Delhi India; ^8^ Apollo Hospital Chennai India; ^9^ Private Practice—RSV Skin and Laser Center Chennai India; ^10^ Calcutta National Medical College and Hospital Kolkata India; ^11^ Grant Medical College and Sir J.J. Group of Hospitals Mumbai India; ^12^ Tata Memorial Cancer Hospital Mumbai India; ^13^ La Roche‐Posay Laboratoire Dermatologique Levallois‐Perret France; ^14^ Post Graduate Institute of Medical Education and Research Chandigarh India

**Keywords:** 2‐MNG (Melasyl), depigmenting, dermocosmetics, melasma, PIH, skin of color, skincare

## Abstract

**Introduction:**

Hyperpigmentation disorders impose a substantial clinical and psychosocial burden in individuals with darker skin phototypes, yet remain underrepresented in dermatological research. In India, where a large proportion of the population reports pigmentary concerns, conditions such as post‐inflammatory hyperpigmentation (PIH), melasma, and periorbital hyperpigmentation significantly affect quality of life. Conventional therapies are often limited by irritation, relapse, safety concerns in skin of color, and poor long‐term adherence, prompting interest in dermocosmetic alternatives with improved tolerability.

**Objective:**

To establish expert consensus on the epidemiology, clinical challenges, and evolving management strategies for hyperpigmentation in India, with particular focus on dermocosmetics and non‐tyrosinase depigmenting agents.

**Methods:**

A modified Delphi consensus was conducted among senior dermatologists from multiple Indian states. Findings from a multinational epidemiological survey (34 countries; 48 000 participants, including 1000 from India) were integrated with a structured pre‐consensus survey assessing clinical patterns and treatment practices. An in‐person advisory board meeting (Jaipur, February 2025) facilitated iterative discussions on disease burden, therapeutic approaches, procedural interventions, and emerging depigmenting agents, including 2‐mercaptonicotinoyl glycine (2‐MNG).

**Results:**

Melasma was the most frequently encountered pigmentary disorder in Indian practice (mean 32%), followed by acne induced PIH (21.5%) and non‐acne PIH (13.1%). Key challenges included treatment resistance, recurrence, irritation, quality of life impairment, procedural downtime, and limited maintenance options. The panel identified 2‐MNG as a promising depigmenting agent with a non‐tyrosinase mechanism and favorable tolerability in darker skin phototypes.

**Limitations:**

Findings are based on expert consensus, existing literature, and limited survey data; larger long term clinical studies in Indian populations are warranted.

**Conclusion:**

Dermocosmetic innovations such as 2‐MNG may address unmet needs in hyperpigmentation management in skin of color, highlighting the need for tailored, evidence‐based strategies.

## 1 Introduction

Hyperpigmentation disorders are among the most common dermatological concerns worldwide, yet remain disproportionately underrepresented in clinical research and evidence‐based treatment guidelines. While individuals with Fitzpatrick skin types IV–VI represent the global majority, the vast majority of dermatological clinical trials, therapeutic agents, and treatment algorithms have been developed and validated predominantly in lighter skin phototypes [1]. This disparity has resulted in a critical knowledge gap regarding the safety, efficacy, and long‐term outcomes of depigmenting therapies in darker‐skinned populations who paradoxically face the highest burden of pigmentary disorders [2].

The unique melanogenic characteristics of skin of color including increased melanocyte density, larger and more widely distributed melanosomes, enhanced tyrosinase activity, and heightened susceptibility to post‐inflammatory pigmentary changes necessitate specialized therapeutic approaches. Furthermore, differences in epidermal barrier function, inflammatory response patterns, and photoprotection requirements underscore the inadequacy of extrapolating treatment protocols directly from studies conducted in lighter skin types. Despite this, comprehensive data on optimal treatment regimens, combination therapies, and long term safety profiles specific to skin of color remain limited.

### 1.1 Clinical and Psychosocial Burden in India

Hyperpigmentation disorders are prevalent skin conditions characterized by patches of skin darkening compared to the surrounding skin. Chronic ultraviolet exposure is the primary etiological factor for solar lentigines and uneven pigmentation [2, 3, 4], whereas hormonal influences, cutaneous injury, acne, and inflammatory dermatoses frequently contribute to PIH, particularly in darker phototypes [5]. In India, hyperpigmentation disorders are widespread due to the tropical climate, intense UV radiation, and high prevalence of Fitzpatrick skin types IV to VI, which demonstrate increased susceptibility to melasma and PIH due to higher melanin content [6].

The psychosocial impact of hyperpigmentation in India is compounded by deep‐rooted cultural stigma associated with darker skin tones and uneven pigmentation. Patients with visible pigmentary disorders frequently report avoiding social interactions, experiencing discrimination, and suffering significant impairment in quality of life often comparable to that seen in other chronic dermatological conditions. This psychological burden underscores the urgent need for safe, effective, and culturally sensitive therapeutic interventions.

### 1.2 Current Treatment Landscape and Limitations

A diverse array of treatment practices exists for managing hyperpigmentation, ranging from topical agents to in clinic procedures. Hydroquinone, retinoids, kojic acid, azelaic acid, and niacinamide are front line treatment options for skin depigmentation in current practices, and many of these topical agents function through the inhibition of the enzyme tyrosinase, which is a key regulator in melanin production [7]. Tyrosinase catalyzes the initial and rate limiting steps of the melanogenesis pathway, specifically the hydroxylation of l‐tyrosine to l‐DOPA, an early precursor in melanin biosynthesis, and the subsequent oxidation of l‐DOPA to dopaquinone. As tyrosinase serves as a strategic checkpoint for modulating melanin production, it has become a key molecular target in the development of topical depigmenting agents [8, 9, 10, 11]. Despite their clinical efficacy, many of these agents cause adverse reactions such as skin irritation and, less frequently, ochronosis, especially after long term use of hydroquinone.

In terms of levels of evidence, hydroquinone 4% remains the gold standard depigmenting agent with the highest level of clinical evidence (Level I–II), supported by multiple randomized controlled trials demonstrating efficacy in melasma and PIH. Tretinoin and combination formulations (e.g., Kligman's triple combination of hydroquinone, tretinoin, and fluocinolone acetonide) similarly carry Level I evidence. Kojic acid, azelaic acid (15%–20%), and niacinamide are supported by Level II–III evidence from randomized and comparative studies. Alpha hydroxy acids (glycolic acid) and botanical agents such as arbutin, licorice extract, and tranexamic acid (topical and oral) carry Level II–III evidence, predominantly from randomized controlled and open label trials. Cysteamine has emerging Level II evidence. This hierarchy of evidence must contextualize treatment selection alongside individual tolerability, skin phototype, and risk of paradoxical hyperpigmentation.

Although hyperpigmentation disorders are recognized as significant dermatological concerns, patients' treatment adherence is often hindered by inconsistent engagement with prescribed regimens, particularly in the absence of immediate visible outcomes. This challenge is exacerbated by the lack of public awareness of the necessity for photoprotection and the adjunctive utility of broad spectrum sunscreens [12]. Therefore, offering treatments that are effective, suitable for long term use, and minimally irritating, while increasing public awareness of sun protection and appropriate treatment selection, are essential for successful hyperpigmentation management.

### 1.3 The Emerging Role of Dermocosmetics

In recent years, dermocosmetics have emerged as a compelling modality for hyperpigmentation, providing more accessible and socially acceptable skincare solutions [13]. These treatments are generally milder, suitable for prolonged use with minimal adverse effects, and tailored to diverse skin phototypes and climatic factors, making them more effective and user friendly [14]. Thus, dermocosmetics bridge aesthetic aspirations and dermatological wellness, promoting a comprehensive and proactive skincare paradigm [15].

Among recent advancements in dermocosmetics, a promising breakthrough is the discovery of 2‐mercaptonicotinoyl glycine (2‐MNG; Melasyl), a potential inhibitor of melanin production. Unlike conventional agents that primarily target tyrosinase, 2‐MNG employs a novel mechanism by intercepting melanin precursors early in the pigmentation cascade without interfering with tyrosinase, thereby potentially curtailing melanin formation before its visible appearance on the skin [16]. Clinical outcomes have shown that 2‐MNG‐containing products can reduce hyperpigmentation associated with melasma and post‐inflammatory hyperpigmentation, enhance skin clarity, and even tone without the adverse effects commonly associated with conventional depigmenting agents such as hydroquinone or retinoids [17, 18, 19]. As dermocosmetics continue to gain credibility among healthcare professionals and consumers, agents like 2‐MNG represent scientifically advanced, well tolerated skincare solutions [20]. However, evidence for the use of 2‐MNG in other hyperpigmented lesions, including solar lentigines, remains limited and warrants further peer‐reviewed clinical investigation.

### 1.4 Research Gap and Clinical Relevance of This Consensus

Despite the high prevalence and significant impact of hyperpigmentation disorders in India, there is a paucity of region specific, evidence based consensus guidelines addressing the unique therapeutic challenges faced by Indian dermatologists. Existing international guidelines often lack applicability to Indian skin types, climatic conditions, treatment access, and socio cultural contexts. Furthermore, emerging therapeutic modalities such as 2‐MNG have not been systematically evaluated by Indian experts in the context of local clinical practice.

This expert consensus addresses these critical gaps by: (1) documenting the epidemiology and quality of life impact of pigmentary disorders specifically in the Indian population; (2) systematically surveying current treatment practices among Indian dermatologists; (3) identifying key clinical and psychosocial challenges in treatment adherence and patient management; (4) consolidating current evidence based treatment practices tailored to Indian skin; and (5) evaluating the potential role of novel depigmenting agents such as 2‐MNG as safer, long‐term alternatives to conventional tyrosinase inhibitors. By providing region‐specific, clinically relevant recommendations grounded in both epidemiological data and expert clinical experience, this consensus aims to improve therapeutic outcomes and patient satisfaction in the management of hyperpigmentation disorders in India.

## 2 Materials and Methods

### 2.1 Study Design

This study employed a modified Delphi consensus methodology integrating three complementary data sources: (1) a multinational epidemiological cross‐sectional survey of pigmentary disorder prevalence and quality of life impact; (2) a structured pre‐consensus survey of Indian dermatologists assessing current treatment practices and clinical challenges; and (3) an in‐person expert advisory board meeting with iterative rounds of deliberation to achieve consensus on key clinical recommendations.

### 2.2 Multinational Epidemiological Survey

A large scale epidemiological cross sectional survey was conducted across 34 countries from December 2022 to February 2023 by L'Oréal Dermatological Beauty. The survey included over 48 000 participants, of whom 1000 were from India. The study assessed the prevalence of six common pigmentary disorders encompassing both hyperpigmentary and hypopigmentary conditions: solar lentigines, post‐inflammatory hyperpigmentation (PIH), melasma, axillary hyperpigmentation and periorbital hyperpigmentation. Participants completed a standardized self‐assessment tool incorporating expert developed textual and visual diagnostic references. These diagnostic references included detailed written descriptions of each condition and photographic examples across different skin phototypes, with specific guidance to differentiate between clinically overlapping entities (e.g., periorbital hyperpigmentation versus periorbital melasma, physiologic versus pathologic axillary hyperpigmentation). Critically, all self‐reported pigmentary disorders were subsequently clinically confirmed by dermatologists using these expert developed diagnostic references to ensure diagnostic uniformity and accuracy. Participants were also asked whether their pigmentary disorder had been previously confirmed by a healthcare professional or dermatologist, providing additional validation of self‐reported diagnoses. The epidemiological findings were therefore interpreted as contextual prevalence estimates rather than definitive diagnostic frequencies. Quality of life impact was assessed using the Dermatology Life Quality Index (DLQI), and stigmatization metrics were collected through validated psychosocial questionnaires [21].

### 2.3 Pre‐Consensus Survey of Indian Dermatologists

As an integral component of the modified Delphi process, a structured pre‐consensus survey was administered to 11 senior dermatologists practicing across different regions of India. This survey was designed to systematically document current clinical practices, treatment approaches, and challenges in managing hyperpigmentation disorders in the Indian population. The survey instrument, developed by the steering committee, included quantitative and qualitative components. Data were collected electronically in advance of the consensus meeting and served as the foundation for structured expert panel discussions. The following survey domains were evaluated to capture real world clinical practice patterns and expert perspectives on hyperpigmentation management:

- *Clinical prevalence assessment*: Respondents estimated the approximate percentage of their patient population presenting with each of the six major pigmentary disorders (melasma, acne‐induced PIH, non‐acne PIH, periorbital hyperpigmentation, axillary hyperpigmentation, solar lentigines).
- *Treatment protocols*: For each pigmentary disorder, respondents documented their first line and second line treatment approaches for both mild and severe forms, including specific active ingredients, combination regimens, and typical duration before clinical reassessment.
- *Procedural interventions*: Utilization patterns of in‐office procedures (chemical peels, laser therapies, microneedling) were documented for each pigmentary disorder.
- *Clinical challenges*: Open ended questions elicited expert perspectives on key therapeutic challenges, including treatment resistance, recurrence rates, adverse effects, patient compliance, and quality of life considerations.
- *Maintenance therapy preferences*: Post‐treatment maintenance regimens and preferred dermocosmetic ingredients were systematically documented.

### 2.4 Consensus Panel Composition

The advisory board meeting was held in Jaipur in February 2025, organized by L'Oréal Dermatological Beauty. The panel comprised 11 senior dermatologists representing different geographic regions including Delhi, Mumbai, Kolkata, Chennai, and Chandigarh. Panelist recruitment was centrally coordinated through direct invitation to recognized experts in clinical dermatology and pigmentary disorders. Selection criteria included: (1) board certification in dermatology; (2) a minimum of 10 years of clinical experience; (3) active clinical practice with substantial patient volume for pigmentary disorders; (4) regional representation to capture geographic diversity in clinical practice patterns; and (5) prior participation in advisory activities or consensus development. There were no public advertisements, secondary nominations, or external referrals, ensuring centralized oversight and consistency in the selection process.

### 2.5 Modified Delphi Consensus Process

The consensus development followed a modified Delphi approach consisting of pre‐meeting preparation, structured in‐person deliberations, and iterative refinement of recommendations. The process unfolded as follows:

- *Phase 1 (Pre‐meeting preparation)*: Panelists were provided with: (a) summary results from the multinational epidemiological survey, including India specific prevalence data and DLQI scores; (b) aggregated findings from the pre‐consensus survey of Indian dermatologists; and (c) a structured literature review on emerging depigmenting agents, with particular focus on 2‐MNG. Panelists were asked to independently review these materials and prepare preliminary assessments.
- *Phase 2 (In‐person consensus meeting)*: The single day advisory board meeting was conducted on February 7, 2025, at the InterContinental Hotel, Jaipur. The meeting followed a pre‐specified agenda organized into thematic modules: (i) epidemiological burden and quality of life impact; (ii) current treatment practices and regional variations; (iii) clinical challenges and unmet needs; (iv) procedural interventions and combination therapies; (v) emerging therapeutic modalities including 2‐MNG; and (vi) recommendations for patient education and photoprotection strategies.
  For each module, coordinators presented background data followed by facilitated group discussion. Coordinators from L'Oréal Dermatological Beauty facilitated the deliberations but did not exercise decision‐making authority, ensuring that final consensus outcomes reflected the independent clinical judgment of the expert panel. Through multiple successive rounds of discussion, areas of agreement were identified and contentious issues were systematically addressed through iterative refinement.
- *Phase 3 (Consensus formulation)*: Following structured deliberation on each module, panelists formulated consensus based insights and recommendations. Consensus was defined as agreement among at least 80% of panelists on key recommendations. Where full consensus was not achieved, divergent perspectives were documented and presented as areas requiring further research.

The consensus meeting was conducted in English, the language of medical practice across India. No additional linguistic or accessibility adaptations were necessary. All discussions were audio recorded (with participant consent) and professionally transcribed for accuracy in documenting consensus recommendations.

### 2.6 Data Analysis

Quantitative data from the pre‐consensus survey (treatment prevalence estimates, assessment timelines) were analyzed using descriptive statistics (means, ranges). Qualitative data from open‐ended survey questions and consensus meeting discussions were thematically analyzed to identify recurring themes, clinical challenges, and areas of expert agreement. Consensus recommendations were synthesized from the iterative deliberations and organized into clinically relevant domains.

## 3 Results

### 3.1 Epidemiology of Pigmentary Disorders: Global and Indian Prevalence

The multinational cross‐sectional epidemiological survey assessed the prevalence of common pigmentary disorders across 34 countries, encompassing approximately 48 000 participants, including about 1000 individuals from India [21]. Overall, 50% of the global cohort reported experiencing at least one pigmentary disorder, whereas 75% of Indian respondents (*n* ≈ 1000) reported at least one pigmentary condition, representing a 50% higher prevalence compared with the global average and placing India among the countries with the highest overall burden of pigmentary disorders.

Substantial disparities were also evident for individual conditions. In the Indian cohort, post‐inflammatory hyperpigmentation (PIH) was reported by 27.3% of participants (*n* = 266) compared with 15.1% globally, while melasma affected 24.9% (*n* = 239) of Indian respondents versus 11.1% worldwide, corresponding to approximately 1.8‐fold and 2.2‐fold higher prevalence, respectively. Periorbital hyperpigmentation and axillary hyperpigmentation were likewise more prevalent in India, affecting 31.7% (*n* = 308) and 31.2% (*n* = 303) of participants, compared with 14.4% and 17.7% globally. Solar lentigines, although common in the Indian population (33.4%, *n* = 325), demonstrated a smaller difference relative to global estimates (28.1%), possibly reflecting demographic variation and differences in ultraviolet exposure patterns. Collectively, these findings highlight a substantially higher prevalence of both localized and generalized pigmentary disorders in the Indian population (Figure 1).

**FIGURE 1:**
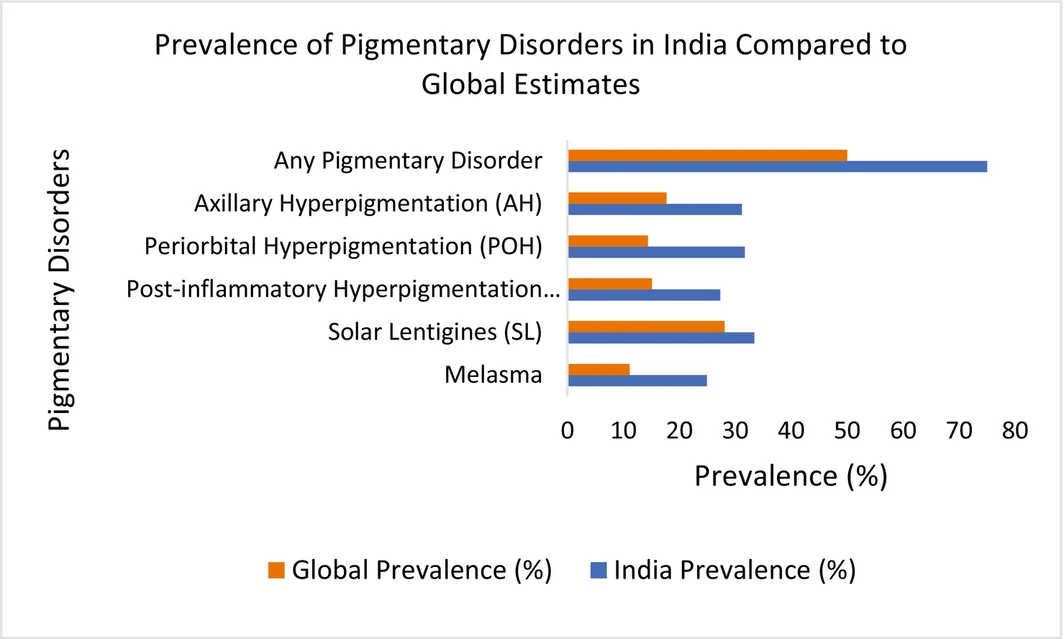
Prevalence of common pigmentary disorders in India compared with global prevalence based on a large multinational epidemiological survey. India shows a consistently higher burden of hyperpigmentation disorders, particularly melasma and post‐inflammatory hyperpigmentation.

Quality of life impairment was markedly elevated among Indian patients with pigmentary disorders. According to the Dermatology Life Quality Index (DLQI), 80% of Indian patients recorded scores exceeding 10, a threshold indicating moderate to severe impairment in quality of life substantially higher than the global average. Stigmatization metrics revealed that 66% of Indian patients avoided social interactions due to pigmentation (vs. 32% globally), 68% avoided family photographs, and 67% felt treated differently during official procedures (Figure 2). These psychosocial impacts underscore the profound burden of hyperpigmentation disorders in the Indian population, extending well beyond cosmetic concerns.

**FIGURE 2:**
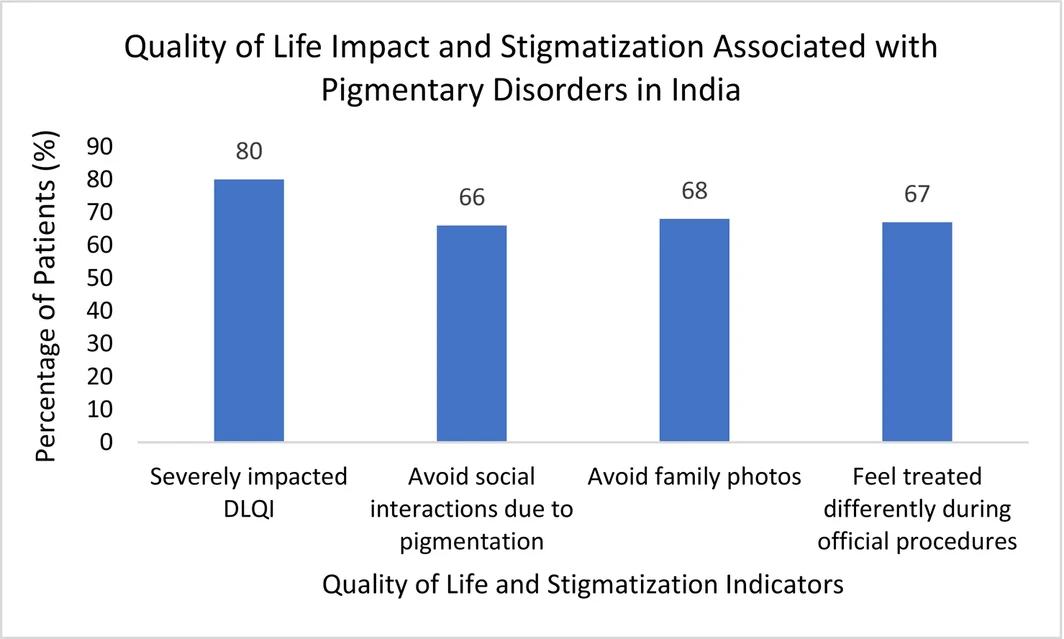
Impact of pigmentary disorders on quality of life and stigmatization among Indian patients. A substantial proportion reported severe Dermatology Life Quality Index impairment and social avoidance behaviors, reflecting significant psychosocial burden.

### 3.2 Clinical Practice Patterns: Pre‐Consensus Survey of Indian Dermatologists

A pre‐consensus survey of 11 senior Indian dermatologists documented the distribution of pigmentary disorders encountered in routine clinical practice (Table 1). Melasma emerged as the most prevalent condition, followed by acne‐induced and non‐acne post‐inflammatory hyperpigmentation. Solar lentigines were least frequently reported, and one respondent identified lichen planus pigmentosus as an emerging concern.

**TABLE 1:** Distribution of pigmentary disorders encountered in routine dermatology practice.

Pigmentary disorder	Mean prevalence (%)	Range (%)
Melasma	32.0	20–50
Acne‐induced PIH	21.5	10–30
Non‐acne PIH	13.1	5–20
Periorbital hyperpigmentation	11.9	5–15
Axillary hyperpigmentation	6.6	3–10
Solar lentigines	2.9	0–5

### 3.3 Treatment Practices for Pigmentation in India

Consensus based treatment patterns for common pigmentary disorders, including third‐line strategies for patients who do not respond to first‐ and second‐line therapy, are summarized in Table 2. First‐line management predominantly involved topical depigmenting agents combined with mandatory photoprotection, while second‐line approaches included chemical peels, laser‐based therapies, microneedling with actives, and selected systemic interventions.

**TABLE 2:** Consensus based treatment practices for common pigmentary disorders in Indian dermatology practice.

Pigmentary disorder	First‐line treatments (topical + mandatory photoprotection)	Second‐line treatments (procedural/systemic)	Third‐line management (after first‐ and second‐line failure)
Melasma	Hydroquinone‐based combinations (HQ 2%–4%) Triple combination cream (HQ 4% + tretinoin 0.05% + fluocinolone acetonide 0.01%) under supervised use ≤ 12 weeks Kojic acid Azelaic acid (15%–20%) Topical tranexamic acid (2%–5%) Topical retinoids Broad‐spectrum sunscreen	Glycolic acid chemical peels (30%–70%) TCA peelsOral tranexamic acid (250 mg twice daily) Microneedling with actives or PRP Q‐switched Nd:YAG laser (low‐fluence toning mode). Non‐ablative fractional lasers	Combination of oral tranexamic acid with energy‐based devices Microneedling with intradermal tranexamic acid delivery Emerging non‐HQ agents (cysteamine 5%, Thiamidol) for HQ‐intolerant patients Picosecond laser for refractory cases Reassess trigger factors and photoprotection adherence
Acne‐induced PIH	Azelaic acid (15%–20%) Glycolic acid (cream or lotion) Topical retinoids; broad‐spectrum sunscreen	Superficial chemical peels (glycolic acid 20%–30%, mandelic acid, salicylic acid) Q‐switched Nd:YAG laser Microneedling with actives	Oral tranexamic acid Non‐ablative fractional lasers Q‐switched Nd:YAG laser Cysteamine cream as adjunct
Non‐acne‐induced PIH	Short‐term low‐potency topical corticosteroids (to address active inflammation) Hydroquinone combinations Azelaic acid (15%–20%) Topical retinoids Broad‐spectrum sunscreen	Superficial chemical peels Q‐switched Nd:YAG laser Microneedling with actives	—
Periorbital hyperpigmentation	Kojic acid Topical vitamin C (10%–20%) Topical vitamin K Botanical lightening agents Low‐concentration retinoids (periorbital use with caution) Broad‐spectrum sunscreen	Superficial chemical peels Platelet‐rich plasma (PRP) Fillers (for vascular or structural component) Laser therapies	—
Axillary hyperpigmentation	Kojic acid Glycolic acid preparations Topical retinoids Keratolytics Broad‐spectrum sunscreen	Superficial chemical peels Laser therapie Microneedling with actives	—
Solar lentigines	Broad‐spectrum sunscreen (primary prevention and adjunct to all treatments) Topical retinoids Non‐steroidal lightening agents (kojic acid, arbutin, topical vitamin C) Broad‐spectrum sunscreen	Q‐switched Nd:YAG laser Intense pulsed light (IPL) Superficial chemical peels Cryotherapy Cautery	—

### 3.4 Clinical Challenges in Hyperpigmentation Management

During the pre‐consensus survey and advisory board discussions, dermatologists identified multiple challenges that complicate the management of hyperpigmentation disorders in routine Indian clinical practice. These challenges were thematically grouped into treatment related, patient related, and systemic factors and are summarized in Table 3.

**TABLE 3:** Summary of clinical challenges encountered in the management of hyperpigmentation disorders.

Category	Key challenges
Treatment related	Treatment resistance; recurrence of pigmentation; irritation from topical therapies; risk of treatment induced post‐inflammatory hyperpigmentation (PIH), particularly in Indian skin; limited effective options for long term maintenance
Patient related	Poor treatment adherence; significant quality of life (QOL) impact; delayed clinical response; persistent or relapsing pigmentation
Systemic/procedural	Patient discomfort associated with procedural downtime; limited tolerance for prolonged or intensive treatment regimens; need for a multimodal therapeutic approach

## 4 Discussion

The integration of multinational epidemiological data, systematic survey of Indian dermatological practice patterns, and expert consensus deliberations provides comprehensive evidence for understanding hyperpigmentation burden in India.

### 4.1 The Discovery and Molecular Mechanism of 2‐Mercaptonicotinoyl Glycine (2‐MNG; Melasyl)

The development of 2‐mercaptonicotinoyl glycine (2‐MNG; Melasyl) represents a paradigm shift in the approach to hyperpigmentation management, emerging from an ambitious artificial intelligence‐powered drug discovery initiative conducted by L'Oréal Research & Innovation. Utilizing advanced *in silico* molecular modeling and high‐throughput virtual screening technologies, researchers systematically evaluated over 100 000 candidate molecules to identify compounds capable of modulating melanogenesis through novel, non‐tyrosinase dependent mechanisms [20]. This computational approach, integrating machine learning algorithms, molecular docking simulations, and predictive ADME (absorption, distribution, metabolism, excretion) modeling, enabled the identification of 2‐MNG as a lead candidate with optimal pharmacological properties including high selectivity for melanin precursors, minimal cytotoxicity, excellent dermal penetration characteristics, and a favorable safety profile.

Conventional depigmenting agents such as hydroquinone, kojic acid, and azelaic acid exert their therapeutic effects primarily through competitive or non‐competitive inhibition of tyrosinase, the rate limiting enzyme in melanogenesis that catalyzes the hydroxylation of l‐tyrosine to l‐DOPA (3,4‐dihydroxyphenylalanine) and the subsequent oxidation of l‐DOPA to dopaquinone. While effective, tyrosinase inhibition can be associated with cytotoxic effects on melanocytes, rebound hyperpigmentation upon treatment discontinuation, and limited long‐term tolerability.

In contrast, 2‐MNG operates through a fundamentally different mechanism that targets the melanogenic cascade *downstream* of tyrosinase activity, without interfering with the enzyme itself. Specifically, 2‐MNG acts as a potent interceptor of key melanin biosynthetic intermediates dopaquinone, 5,6‐dihydroxyindole (DHI), and 5,6‐dihydroxyindole‐2‐carboxylic acid (DHICA), which are normally polymerized to form eumelanin (brown black pigment) and pheomelanin (red yellow pigment). Through covalent binding to these reactive quinone intermediates, 2‐MNG prevents their incorporation into polymeric melanin structures, effectively attenuating pigment production without disrupting melanocyte viability or tyrosinase function [20]. This mechanistic divergence confers several theoretical advantages: (1) preserved melanocyte integrity and function, reducing risk of permanent depigmentation‐like changes; (2) balanced inhibition of both eumelanin and pheomelanin synthesis, potentially offering more uniform depigmentation; (3) lower propensity for rebound hyperpigmentation upon treatment cessation; and (4) improved long‐term safety profile suitable for maintenance therapy.

### 4.2 Clinical Evidence for 2‐MNG: Efficacy and Safety in Darker Skin Phototypes

A comprehensive dermocosmetic formulation was developed for clinical evaluation to target hyperpigmentation disorders, including post‐inflammatory hyperpigmentation, melasma, and solar lentigines, by incorporating 0.5% 2‐MNG alongside complementary adjunctive ingredients: 10% niacinamide (anti‐inflammatory, barrier enhancing, and mild depigmenting properties), *Cystoseira tamariscifolia* extract (marine antioxidant), lipo‐hydroxy acid (LHA; gentle exfoliant), carnosine (antioxidant dipeptide), retinyl palmitate (vitamin A derivative), dipotassium glycyrrhizate (anti‐inflammatory licorice extract), hyaluronic acid (hydration), and glycerin (humectant). This 2‐MNG containing serum was designed to address multiple pathophysiological mechanisms underlying hyperpigmentation while maintaining excellent tolerability for long term use.

#### 4.2.1 Comparative Efficacy Study: 2‐MNG Versus 4% Hydroquinone (Brazil)

A pivotal randomized, investigator‐blinded comparative clinical trial conducted in Brazil evaluated the efficacy and tolerability of the 2‐MNG containing serum versus 4% hydroquinone, the current gold standard depigmenting agent for melasma. The study enrolled women with moderate to severe facial melasma (Fitzpatrick skin types III–V) and utilized the Modified Melasma Area and Severity Index (mMASI) as the primary efficacy endpoint, supplemented by instrumental colorimetry (ITA‐ Individual Typology Angle), clinical photography, and patient‐reported outcomes. Results demonstrated non‐inferiority of the 2‐MNG containing serum to 4% hydroquinone in reducing mMASI scores over 12 weeks, with comparable improvement in skin tone evenness and pigment intensity. Critically, the 2‐MNG formulation exhibited superior tolerability, with significantly lower incidence of cutaneous irritation (erythema, burning, stinging), desquamation, and discomfort compared to hydroquinone. Furthermore, sustained improvement was observed through 24 weeks, suggesting durable therapeutic benefit [18]. These findings position 2‐MNG as a viable alternative to hydroquinone for patients requiring prolonged treatment or those who experience intolerance to conventional tyrosinase inhibitors.

#### 4.2.2 Non‐Inferiority Study: 2‐MNG Versus 5% Cysteamine (India)

A parallel clinical investigation conducted in India compared the 2‐MNG‐based serum against 5% cysteamine cream, an emerging alternative depigmenting agent with demonstrated efficacy in melasma but limited by odor and potential irritation. The study enrolled Indian women with melasma (predominantly Fitzpatrick types IV–V) and employed mMASI as a primary endpoint. Results confirmed non‐inferiority of 2‐MNG to cysteamine in reducing mMASI scores, while demonstrating superior improvement in investigator assessed and patient reported severity grades. Notably, the 2‐MNG formulation was associated with higher treatment satisfaction scores, attributable to its elegant cosmetic properties (non‐odorous, rapid absorption, non‐greasy finish) and lower irritation potential [22]. These India specific findings validate the efficacy and acceptability of 2‐MNG in the target population and reinforce its suitability for incorporation into Indian dermatological practice.

#### 4.2.3 Post‐Inflammatory Hyperpigmentation: Efficacy in Acne Related Dyschromia

An open label prospective study evaluated the 2‐MNG containing serum in 32 subjects with mild active acne and moderate to severe post‐acne hyperpigmentation (defined as Post‐Acne Hyperpigmentation Index [PAHPI] > 10). Subjects applied the serum twice daily for 12 weeks alongside standard acne therapy. Primary outcomes included change in PAHPI score, instrumental colorimetry (ITA, Δ*E*), and stigmatization assessment using a validated psychosocial questionnaire. Results demonstrated statistically significant improvement in PAHPI scores (mean reduction > 40% from baseline), accompanied by measurable increases in skin luminosity (ITA) and overall color correction (Δ*E*). Importantly, reduction in stigmatization scores underscored the meaningful psychosocial benefit of effective PIH treatment [23]. The formulation was well tolerated without exacerbation of underlying acne or induction of new inflammatory lesions, supporting its use as adjunctive therapy in acne management protocols.

A double blind, randomized, head to head comparative trial conducted in Mauritius evaluated the 2‐MNG containing serum against a thiamidol containing serum (another novel non‐tyrosinase depigmenting agent) combined with broad spectrum SPF 50+ sunscreen in the management of PIH. Over 12 weeks, the 2‐MNG formulation demonstrated superior efficacy in reducing total PAHPI score, with significantly greater and faster improvement in lesion count subscore at all assessment timepoints (weeks 4, 8, 12). Additionally, 2‐MNG‐treated subjects exhibited superior gains in skin luminosity and ITA at 12 weeks compared to the thiamidol arm [24]. These findings suggest that 2‐MNG may offer enhanced efficacy in PIH compared to other emerging depigmenting agents, warranting further comparative studies.

### 4.3 Safety Profile and Tolerability Considerations

Across all clinical studies conducted to date, 2‐MNG containing formulations have demonstrated an excellent safety profile with no evidence of melanocyte cytotoxicity, confetti‐like leukoderma‐like permanent depigmentation adverse effects occasionally associated with prolonged hydroquinone use. Pharmacokinetic assessments conducted on representative formulations detected no measurable systemic absorption of 2‐MNG following topical application, suggesting negligible systemic exposure and correspondingly low risk of systemic toxicity. Cutaneous tolerability was consistently rated as excellent or good, with adverse event rates (mild erythema, transient stinging) comparable to vehicle controls and significantly lower than hydroquinone comparators [25].

However, several important safety limitations warrant acknowledgment. First, clinical trial durations have been limited to 12–24 weeks; long term safety data extending beyond 6–12 months are not yet available and represent a priority for post marketing surveillance. Second, 2‐MNG has not been evaluated in pregnant or lactating women extensively, and its use in these populations cannot currently be recommended pending dedicated safety studies. Third, data on potential for tachyphylaxis (loss of efficacy with prolonged use) and pigmentation recurrence patterns following treatment discontinuation remain incomplete. Fourth, while tested extensively in Southeast Asian and South American populations with Fitzpatrick types III‐V, additional studies in sub‐Saharan African skin (type VI) and in pediatric populations are needed to establish safety and efficacy across the full spectrum of skin of color.

### 4.4 Expert Consensus Perspectives on 2‐MNG Integration in Indian Dermatological Practice

The expert panel expressed strong interest in 2‐MNG‐based products as valuable additions to the therapeutic armamentarium for hyperpigmentation management in India, particularly for scenarios where conventional agents are suboptimal. Specific clinical applications identified as promising include:

- *Long term maintenance therapy*: Following initial intensive treatment with hydroquinone or combination regimens, 2‐MNG‐based products may serve as safer, better tolerated maintenance agents to prevent recurrence while minimizing cumulative exposure to cytotoxic depigmenting agents.
- *Alternative first‐line therapy for hydroquinone intolerant patients*: Patients experiencing irritation, contact dermatitis, or subjective intolerance to hydroquinone may benefit from 2‐MNG as an efficacious alternative with a superior tolerability profile.
- *Adjunctive therapy in multimodal treatment paradigms*: 2‐MNG formulations may be integrated with procedural interventions (chemical peels, laser therapies, microneedling) and systemic agents (oral tranexamic acid) to optimize outcomes in refractory melasma and severe PIH cases.
- *Post‐procedure pigmentation prophylaxis*: Given the high risk of post‐inflammatory hyperpigmentation following ablative procedures in Indian skin, prophylactic use of well‐tolerated depigmenting agents such as 2‐MNG may mitigate this complication.
- *Combination with photoprotection for sensitive patients*: The non‐irritating profile of 2‐MNG makes it suitable for combination with high SPF sunscreens and antioxidant formulations in patients with sensitive skin or atopic backgrounds who cannot tolerate more aggressive depigmenting regimens.

With rising awareness of skin health, dermatologists increasingly incorporate dermocosmetics for hyperpigmentation management. 2‐MNG (Melasyl) introduces a novel non‐cytotoxic mechanism discovered through AI‐powered screening of > 100 000 compounds. Unlike conventional tyrosinase inhibitors, 2‐MNG intercepts melanin precursors (dopaquinone, DHI, DHICA) without inhibiting tyrosinase activity, enabling balanced pigment modulation while mitigating cytotoxicity risks.

Clinical studies in Brazil and India demonstrated non‐inferiority to 4% hydroquinone and 5% cysteamine respectively, with superior tolerability. 2‐MNG has been tested on Southeast Asian skin types (Fitzpatrick III–V) with no safety concerns, reinforcing suitability for Indian populations.

### 4.5 Clinical Perspectives on 2‐MNG‐Based Solutions in India

There is growing recognition of the therapeutic potential of 2‐MNG‐containing products, especially as a safer alternative to hydroquinone for extended use directly addressing one of the key unmet needs identified in the pre‐consensus survey. Experts emphasized the need for more long‐term studies specifically tailored to the Indian population to reinforce its therapeutic efficacy across diverse pigmentation subtypes, skin phototypes, and climatic conditions. 2‐MNG‐containing products were identified as promising adjuncts in multimodal treatment paradigms, particularly alongside lasers, chemical peels, and oral tranexamic acid, to optimize therapeutic outcomes in stubborn pigmentation cases.

Furthermore, dermatologists acknowledged the potential utility of 2‐MNG‐based products as maintenance therapy following hydroquinone regimens, where rebound hyperpigmentation and cutaneous irritation are common concerns. This application directly addresses the challenge of limited safe and effective long‐term maintenance options identified in the clinical practice survey. When recommending pigmentation targeted skincare, Indian dermatologists emphasized the importance of formulations offering effective photoprotection, facilitating post‐treatment maintenance, non‐irritating properties, fragrance free composition, and dermatological testing criteria that align well with the formulation profile of 2‐MNG‐containing products.

Experts emphasized the need for additional research to reinforce dermatologists' confidence in 2‐MNG‐based formulations and address remaining uncertainties. Critical research priorities identified included: (1) determining whether pigmentation recurs following treatment discontinuation and the typical timeframe for such recurrence; (2) establishing whether therapeutic effects are reversible or permanent, which has important implications for patient counseling; (3) evaluating the potential for secondary loss of efficacy or tachyphylaxis with prolonged use; (4) characterizing non‐responders and analyzing contributing factors to guide patient selection; (5) conducting head‐to‐head comparisons with hydroquinone (the established gold standard) in Indian populations; (6) performing region‐specific studies with adequate sample sizes to enhance clinical relevance; and (7) analyzing differential responses based on melasma chronicity (longstanding versus recent onset) and subtype (epidermal, dermal, mixed, vascular components).

The consensus aligned with established clinical practices while emphasizing the role of emerging agents such as 2‐MNG (Melasyl) as valuable alternatives or adjuncts to traditional tyrosinase inhibitors, particularly for patients requiring long‐term therapy or those intolerant to conventional agents.

### 4.6 Emerging Non‐Hydroquinone Depigmenting Agents

Beyond 2‐MNG, several non‐hydroquinone agents have accruing evidence in skin of color and should be considered within this therapeutic landscape. Cysteamine 5% is an antioxidant and tyrosinase inhibitor; comparative data versus HQ 4% and triple combination are available [26, 27, 28], including a brief report in skin of color [29]. Thiamidol (isobutylamido thiazolyl resorcinol) is a topical tyrosinase inhibitor with preliminary efficacy and safety data in melasma in skin of color [29]. All of these agents, alongside 2‐MNG, are recognized in the international Delphi consensus by Sarkar et al. (JEADV 2025, Statement 25, High consensus) [30] as viable options for patients intolerant to first‐line agents or moving to maintenance. The mechanisms, evidence, and clinical use of the three principal emerging agents are summarized in Table 4.

**TABLE 4:** Emerging non‐hydroquinone depigmenting agents: Mechanisms, evidence, and clinical use.

Agent	Mechanism of action	Evidence cited in Sarkar et al., Statement 25	Clinical use	Safety concerns raised from literature
Cysteamine 5%	Antioxidant and tyrosinase inhibitor [26]	High consensus [30] Lima et al. [26]: cysteamine 5% versus HQ 4% Sarkar et al. [29]: brief report in skin of color	Alternative for HQ‐intolerant patients or in maintenance phase; comparative efficacy demonstrated versus HQ 4%	May promote phaeomelanin [27]
Thiamidol (isobutylamido, thiazolyl, resorcinol)	Selective topical tyrosinase inhibitor [28]	High consensus Sarkar et al. [30]: preliminary report in melasma, skin of color	Alternative for HQ‐intolerant patients or in maintenance phase; evidence limited to a preliminary report in skin of color	Cases of contact dermatitis reported [10, 31, 32]
2‐MNG (2‐mercaptonicotinoyl glycine)	Inhibits immediate darkening and new melanin synthesis via covalent conjugation of the thiol group of 2‐MNG to melanin precursors [17]	High consensus [30] de Dormael et al. [17]: 2‐MNG prevents UV‐induced skin darkening Note: Therapeutic trial evidence is from MelaB3 formulation [18, 22]; no standalone 2‐MNG RCT available	Alternative for HQ‐intolerant patients or in maintenance phase	

## 5 Limitations

This consensus has several important limitations that warrant acknowledgment. First, the pre‐consensus survey of Indian dermatologists included only 11 respondents from selected urban centers, which may not fully represent the diversity of dermatological practice across India's varied geographic, socioeconomic, and healthcare settings. Rural and semi‐urban practice patterns, resource constraints, and patient demographics may differ substantially from those captured in this survey. Second, treatment prevalence estimates provided by surveyed dermatologists represent subjective clinical impressions rather than systematic chart reviews or prospective data collection, introducing potential recall bias and estimation error.

Third, while the multinational epidemiological survey provided valuable prevalence data, the self‐reported nature of pigmentary disorder assessment despite clinical confirmation using diagnostic references may have introduced classification bias, particularly for conditions with overlapping presentations. Fourth, the consensus process involved a single in‐person meeting, limiting the opportunity for extensive iterative refinement typical of classical Delphi methodologies. Fifth, the advisory board was organized by L'Oréal Dermatological Beauty, manufacturer of 2‐MNG‐containing products, introducing potential sponsor bias despite efforts to ensure independent expert judgment.

Therapeutic evidence for 2‐MNG in melasma and post‐inflammatory hyperpigmentation derives from studies of a 2‐MNG containing serum. Standalone 2‐MNG has been evaluated in a Bayesian network meta‐analysis of UV‐induced pigmentation [20], in which it ranked first among 14 tested molecules, supporting its intrinsic activity; *the 2‐MNG containing serum has been formulated to target the different pathophysiology actors of pigmentary disorders including melanin synthesis, inflammation, exfoliation, epidermal renewal, oxidative stress*, *and skin barrier protection. Clinical studies have been performed in different skin conditions such as melasma and acne‐induced PIH to prove its efficacy and tolerability*. Additional long‐term, randomized controlled clinical trials specifically conducted in diverse Indian populations (representing different geographic regions, age groups, pigmentation subtypes, and skin phototypes) are necessary to further validate the efficacy, safety, optimal dosing, combination strategies, and long‐term outcomes of 2‐MNG in the Indian context.

## 6 Conclusion

This expert consensus integrates multinational epidemiological data, systematic documentation of Indian dermatological practice patterns, and structured expert deliberations to provide a comprehensive understanding of hyperpigmentation management in India. The findings underscore the disproportionate burden of pigmentary disorders in the Indian population, with prevalence rates substantially exceeding global averages and profound quality of life impairment. The systematic survey of current treatment practices reveals the complexity of therapeutic decision making, the essential role of multimodal approaches combining topical agents and procedural interventions, and the critical unmet need for safe, effective, well‐tolerated agents suitable for long term maintenance therapy.

Hyperpigmentation disorders disproportionately affect populations with darker skin phototypes, yet remain significantly underrepresented in dermatological research and evidence based therapeutic guidelines. This consensus addresses critical gaps by documenting region specific epidemiology, systematically characterizing current clinical practices and challenges, and evaluating emerging therapeutic modalities tailored to skin of color. The identification of key clinical challenges including treatment resistance, recurrence, ochronosis risk, irritation, limited long term options, unrealistic patient expectations, poor photoprotection adherence, and profound psychosocial impact provides a roadmap for targeted interventions to improve therapeutic outcomes.

The emergence of novel molecules such as 2‐MNG (Melasyl), supported by promising clinical outcomes in diverse populations including Indian cohorts, represents a meaningful advancement in the therapeutic armamentarium. The non‐tyrosinase targeting mechanism, favorable safety profile demonstrated in darker skin phototypes, and potential for long term use position 2‐MNG as a valuable alternative or adjunct to conventional depigmenting agents, particularly for maintenance therapy where hydroquinone use is limited by safety concerns. Nevertheless, further long term, region specific research with adequate sample sizes is necessary to validate its efficacy across diverse skin types, pigmentation subtypes, combination treatment regimens, and to establish optimal treatment algorithms.

Collaborative expert consensus, continued investment in skin of color research through adequately powered clinical trials in diverse populations, development of culturally sensitive patient education initiatives addressing photoprotection and realistic treatment expectations, and formulation of evidence‐based treatment protocols tailored to Indian dermatological practice will continue to refine therapeutic strategies and improve clinical outcomes for patients with hyperpigmentation disorders in India and globally.

## Author Contributions

Mukta Sachdev contributed to the interpretation of the literature, formulation of consensus statements, and critical review of the manuscript for intellectual content. Delphine Kerob contributed to the critical review of the manuscript for intellectual content. Neena Khanna, Niti Khunger, Sushil Tahiliani, Anil Ganjoo, Nina Madnani, Maya Vedamurthy, Abhishek De, Rajesh Kumar, and Davinder Prasad reviewed the manuscript content for scientific merit and coherence. All authors contributed to the expert consensus process and the review of the manuscript, and all authors read and approved the final manuscript.

## Funding

This advisory board meeting and consensus development process were organized and funded by L'Oréal Dermatological Beauty, the manufacturer of 2‐MNG (Melasyl) and MelaB3 serum.

## Ethics Statement

This work is based on expert consensus.

## Consent

Written informed consent was obtained from all expert participants involved in the pre‐consensus survey and advisory board meeting for their voluntary participation and for the use of the anonymized information and opinions provided during the consensus process. No individual patient data or identifiable patient information were collected or reported in this study. Therefore, patient consent was not applicable.

## Conflicts of Interest

All expert panelists received honoraria for their participation in the advisory board meeting. The multinational epidemiological survey discussed in this consensus was conducted by L'Oréal Dermatological Beauty. While the meeting was facilitated by L'Oréal representatives, all clinical assessments, expert deliberations, and consensus recommendations reflect the independent professional judgment of the participating dermatologists. The authors acknowledge this potential conflict of interest and affirm that the scientific integrity of the consensus process was maintained throughout. The decision to publish and the content of this manuscript were determined solely by the expert panel. Readers should note that clinical data on 2‐MNG (Melasyl) discussed in this manuscript were provided by L'Oréal Dermatological Beauty, the manufacturer of the product; independent peer‐reviewed publications are cited where available, and the panel's recommendations were formulated on the basis of their independent clinical judgment.

## Data Availability

The data supporting the findings of this study are available from the corresponding author upon reasonable request.
